# Using a Sober Curious Framework to Explore Barriers and Facilitators to Helping Sexual Minority Women Reduce Alcohol-Related Harms: Protocol for a Descriptive Study

**DOI:** 10.2196/63282

**Published:** 2025-03-03

**Authors:** Tonda L Hughes, Lauren Bochicchio, Laurie A Drabble, Belinda Lunnay, David Whiteley, Jillian R Scheer, Beth Meadows, Paul Ward, Carol Emslie

**Affiliations:** 1 Center for Sexual and Gender Minority Health Research School of Nursing Columbia University New York, NY United States; 2 Department of Psychiatry Columbia University New York, NY United States; 3 San Jose State University San Jose, CA United States; 4 Torrens University Adelaide Australia; 5 Glasgow Caledonian University Glasgow United Kingdom; 6 University of Rhode Island Kingstown, RI United States; 7 Torrens University Australia Adelaide Australia

**Keywords:** sexual minority women, drinking, sober curiosity, women, sober, minority, alcohol, protocol, barriers, facilitators, disparities

## Abstract

**Background:**

Globally, women consume less alcohol than men, but alcohol consumption among women has declined less in recent years than among men. Drinking rates and alcohol-related harms vary substantially across population groups of women, and sexual minority women (eg, lesbian, bisexual, and queer) are at notably high risk. An emerging body of literature suggests that in addition to minority stress (eg, stigma, discrimination), drinking norms and drinking cultures likely influence sexual minority women’s drinking. Almost no research has explored these factors as possible targets of interventions. Sober curiosity is a rapidly growing wellness movement that may be particularly salient for sexual minority women. It encourages individuals to be “curious” about the reasons they choose to drink and alcohol’s effects on their life and health.

**Objective:**

The aims of this research are to (1) explore the perspectives of the drinking social worlds of sexual minority women, their awareness of the sober curious movement, perceptions of their own and their peers’ drinking and desire to drink less, and perceived barriers and facilitations to changing their drinking behaviors and (2) identify key elements of an alcohol reduction intervention tailored for sexual minority women.

**Methods:**

We conducted a comprehensive review of the literature on alcohol interventions with sexual minority women. The handful of studies we found paid scant attention to drinking cultures, normative beliefs, or other key elements of sober curiosity. To address the study aims, we are conducting 2 descriptive studies with adult (>18 years) sexual minority women using mixed methods. One includes focus group interviews (n=24-36) and a national survey (n=100-120) with sexual minority women in Scotland. The other includes in-depth interviews (n=18-20) with sexual minority women in the United States. Data from the 2 countries and 3 sources will be analyzed using qualitative and quantitative methods to identify patterns and relationships across data to validate or corroborate findings.

**Results:**

Each of the studies received ethics approval in August 2023 and is currently open for recruitment. We anticipate completing data collection in spring 2025. The results of qualitative analyses will be summarized as themes, and results of survey data analyses will be summarized in tables. Findings will be presented to 2 panels of international experts who will assist in identifying critical elements of an alcohol reduction intervention tailored to the unique needs of sexual minority women.

**Conclusions:**

With the assistance of the expert panels, we will use Acceptability, Practicability, Effectiveness, Affordability, Side-Effects, and Equity criteria to inform the development of a tailored intervention building on tenants of sober curiosity to assist sexual minority women in reducing harmful drinking.

**International Registered Report Identifier (IRRID):**

DERR1-10.2196/63282

## Introduction

### Background

#### Overview

Hazardous alcohol consumption is a pattern of alcohol use that increases the risk of harmful consequences [1]. As a leading risk factor for poor health outcomes worldwide, hazardous drinking is a global public health concern. According to the World Health Organization (WHO) [2], heavy drinking is a causal factor in more than 200 disease and injury conditions; it is also among the top 4 risk factors highlighted in the WHO global strategy for prevention and control of noncommunicable diseases. Efforts to reduce hazardous drinking are a major challenge because in many parts of the developed world, consuming alcohol at risky levels is widely socially acceptable and oftentimes viewed as preferable to abstinence, particularly within social worlds structured around heavy drinking norms [3].

The 2018 WHO Global Status Report on Alcohol and Health [4] suggests that women’s drinking has decreased in most countries. However, this is not the case in some Western high-income countries including the United States [5], the United Kingdom [6], and Australia [7]. An increase in women’s drinking is among the most prominent trends in alcohol consumption in the United Kingdom in recent history [8]. Further, alcohol consumption patterns and alcohol-related harms are not evenly distributed across population groups of women [9-12]. As described by Bloomfield's [13] “alcohol harm paradox", although different groups of women may drink similar quantities of alcohol, they often experience differential harms based on their level of social disadvantage. It is important to understand the contemporary determinants of health that contribute to hazardous drinking and make it more (or less) difficult for socially marginalized women to reduce alcohol consumption, particularly when alcohol is readily accessible and is used to facilitate or form social connections. In this paper, we review the literature regarding existing alcohol interventions for sexual minority women (eg, lesbian, bisexual, and queer), discuss barriers and facilitators to interventions that support sexual minority women who wish to reduce their alcohol use, and describe 2 studies, one in Scotland and one in the United States, that aim to support the development of an intervention designed to reduce alcohol-related harms among sexual minority women.

#### Sexual Orientation–Related Disparities in Alcohol Use

Hazardous drinking is among the most prominent health-related disparities when comparisons of heterosexual and sexual minority women are undertaken globally [14-16]. For example, findings from a US national sample found that compared to heterosexual women, sexual minority women were nearly 4 times as likely to engage in heavy episodic drinking [17]. A population-based study conducted in England found that lesbian, gay, and bisexual (LGB) adults (32%) were more likely than heterosexual adults (24%) to drink at harmful levels [18]. This sexual orientation–related disparity is hypothesized to be due to several factors: (1) minority stress related to discrimination and marginalization that impacts mental health and contributes to using alcohol as a coping strategy [14,19-21]; (2) sexual minority women are more likely than heterosexual women to have experienced lifetime trauma, including childhood sexual abuse, childhood physical abuse, adult sexual assault, and intimate partner violence [22-27], that also contributes to alcohol use as a means of coping [28]; (3) the predominantly alcohol-driven nature of the commercial lesbian, gay, bisexual, transgender, queer, and other sexual and gender minority (lesbian, gay, bisexual, transgender, queer or questioning [LGBTQ+]) scene, which facilitates connections and provides a key social environment for many LGBTQ+ individuals and communities [29,30]; and (4) the important role that alcohol plays in identity formation for LGBTQ+ people [14,30,31].

An emerging body of literature suggests that heavy drinking norms also contribute to the heavier drinking patterns of sexual minority women [32]. Drinking is primarily a social activity, often a central part of social occasions in a “social world” where members come together around something they have in common, such as an occupation or an identity [3]. In addition, research suggests that sexual minority women perceive the expectation to drink to be higher in LGBTQ+ spaces than in other contexts [33]. Understanding the drinking norms and drinking behaviors of sexual minority women is essential to informing the development of effective interventions to reduce heavy drinking and alcohol-related harms.

#### Alcohol Treatment and Sexual Minority Women

In the general population, women are less likely than men to seek help related to their drinking [34-36]. Women who drink heavily report experiencing gendered stigma that negatively impacts their desire or ability to access mainstream alcohol treatment services and their experiences when help is sought. Such stigma may be particularly salient for sexual minority women who are already stigmatized based on their sexual identity [35,36]. For example, Dimova et al [37] found that sexual minority women (and sexual minority men) who accessed alcohol treatment were rarely asked about their sexual or gender identity, which precluded exploration of how these factors might impact their drinking or desire to reduce drinking. Both lesbian and bisexual women also reported experiencing heterosexist assumptions (eg, that they were partnered with a man) and feeling judged because of their drinking. Moreover, one recent study using data from the 2020 US National Survey of Substance Abuse Treatment Services found that only 2.4% of substance use treatment facilities offered LGBTQ+-tailored programming [38].

Compared to intervention studies aimed at reducing hazardous drinking among women in the general population, those that focus on the experiences of sexual minority women are more recent and more limited [14,39,40]. The small body of literature on interventions designed to reduce drinking among sexual and gender minority populations has focused predominately on sexual minority men [14,41]. A few studies have been conducted on sexual minority women’s perceptions and experiences of alcohol treatment in the United States, but nearly all have focused on formal treatment or 12-step programs such as Alcoholics Anonymous (AA) [42-48]. Interventions that might be appropriate for heavy-drinking sexual minority women who are not accessing services or for whom alcohol use is not yet problematic enough to warrant formal treatment—but likely to worsen without early intervention—have not been explored.

Many alcohol treatment programs, especially 12-step programs such as AA, emphasize powerlessness, character defects, and making amends to those harmed by one’s drinking—features that may be off-putting for sexual minority women who experience stigma, discrimination, and rejection based on their gender and sexual minority status. While AA is not affiliated with a religious organization, it uses a framework based on religious spirituality. Sexual minority women who do not identify with a particular religion or who have had negative experiences with religious institutions are less likely to access AA [43]. Access to nonreligious and affirming alternatives to AA is important, given research documenting low rates of endorsing religiosity and an absence of a protective effect of religiosity on heavy drinking among sexual minority women relative to heterosexual women [49,50]. Sexual minority women and sexual minority men are more likely than their heterosexual counterparts to express interest in accessible, nonstigmatizing alternatives to AA and traditional treatment approaches [51]. Sober curiosity, described in detail in “Potential Usefulness of an Intervention Based on Sober Curiosity” section, is an identity-affirming approach to alcohol reduction that offers such an alternative.

#### Existing Evidence Regarding Alcohol Interventions With Sexual Minority Women

In a large global scoping review of studies focused on alcohol and other drug use among sexual minority women, Hughes et al [14] describe the dearth of research related to substance use interventions as “a gaping hole.” Although studies of alcohol treatment effectiveness sometimes report findings for sexual minority participants, they rarely report findings specific to sexual minority women. For example, Zajac et al [52] used data from 5 randomized clinical trials to compare the effectiveness of contingency management with financial incentives versus standard intensive outpatient care treatment among 920 LGB and heterosexual participants with a substance use disorder. Differences between LGB and heterosexual participants were compared across 3 substance use outcomes: treatment retention, the longest duration of abstinence, and percent negative substance use screens. Contingency management plus intensive outpatient treatment did not show statistically significant differences for any of the 3 outcomes. However, the LGB group means were higher than those of heterosexual participants for treatment retention and the longest duration of abstinence. Although not strong enough to reach statistical significance, these differences were considered potentially clinically significant. Unfortunately, analyses aggregated LGB participants, which prohibited specific examination of outcomes among sexual minority women.

In another study that combined sexual (and gender) minority participants, Gilmore et al [53] evaluated Positive Change (+*Change*) with 24 undergraduate students from a large university in the Southwestern United States. Of the 24 participants, most (n=17) identified as cisgender heterosexual; 7 identified as sexual minority, gender minority, or both sexual and gender minority. The intervention included content from an integrated alcohol and sexual assault risk reduction program developed for women and a web-based adaptation of a brief motivational interviewing personalized feedback protocol. *+Change* had high usability and acceptability ratings among both cisgender heterosexual and sexual and gender minority participants. Differences based on sexual and gender identity were not examined because of the small sample size.

In the only intervention study undertaken with sexual minority women prior to 2020, Fals-Stewart et al [54] evaluated the efficacy of behavioral couples therapy plus individual therapy compared to individual-based therapy only among lesbian and gay couples, in which at least 1 partner had an alcohol use disorder. Treatment intervention components included developing recovery contracts with partners, teaching partners communication strategies and strategies for reducing triggers and exposure to alcohol, and increasing shared activities. Results indicated that among lesbian couples, those who received the behavioral couples therapy condition reported fewer heavy drinking days at 12-month follow-up compared to those receiving individual therapy only.

Pachankis et al [55] conducted a small (N=60) sexual minority women–adapted cognitive behavioral therapy (CBT) intervention that focused primarily on mental health. This waitlist-controlled trial, Empowering Queer Identities in Psychotherapy, was designed to help sexual minority women understand emotion-driven behavior, reduce emotional avoidance, and learn behavioral skills and other resilience-building strategies. Pachankis et al [55] found that Empowering Queer Identities in Psychotherapy demonstrated preliminary efficacy in reducing sexual minority women’s depression, anxiety, emotion dysregulation, and rumination in the intervention compared to the waitlist control group. The effects of alcohol use were marginally significant, with the immediate intervention group outperforming the waitlist control group. Minority stress responses were not significantly affected by the intervention. In line with previous research, findings suggested that greater attention to sexual minority women’s elevated rates of trauma and hazardous drinking is needed to enhance CBT interventions for sexual minority women.

Boyle et al [56] examined the feasibility and efficacy of reducing alcohol-related risks using personalized normative feedback delivered within a digital competition designed to challenge negative stereotypes and norms about sexual minority women’s drinking. This study was the first to find that activities aimed at correcting sexual minority women–specific drinking and coping norms via personalized normative feedback reduced alcohol consumption among participants receiving this condition compared to those in the control condition who received information about nonsexual minority–specific topics.

In a study conducted with Australian same-sex attracted women, Bush et al [57] used SMS text messaging to examine the feasibility, acceptability, and efficacy of an alcohol intervention called the Step One program. In total, 97 same-sex attracted women who met the criteria for hazardous drinking by scoring ≥8 on the Alcohol Use Disorders Identification Test were randomly assigned to the Step One condition (n=47; mean age 37 years old)—which included a variety of SMS text messages about the health benefits of reducing alcohol intake—or a control condition of messages containing a link to a website with health information and support services for LGBTQ+ people (n=50; mean age 34 years). Participants in both conditions showed significant reductions in alcohol consumption and improved well-being over 4 weeks. The frequency of help-seeking was low; only 4 intervention group participants and 3 control group participants sought help. In total, 10 participants in the intervention group were interviewed about acceptability. Overall findings indicated that the intervention needed to be revised prior to its implementation.

Except for the study by Bush et al [57], all the intervention studies described earlier were conducted in the United States. Although a variety of approaches were used (eg, personalized normative feedback, couples therapy, and CBT), they were all based on individual behavior change models, which have limited effectiveness for heavier drinkers [58]. Efforts that target individual drinkers typically ignore the fact that most drinking is social, occurring in the company of others, and that how much one drinks is influenced by others (eg, pressure to drink more or less).

In summary, sexual minority women have seldom been the focus of intervention studies. When included as part of LGBTQ+ samples, outcomes are rarely reported separately by gender and sexual identity. Consequently, given this population’s social and structural marginalization, it is unknown whether existing treatment options are suitable for sexual minority women. Because the drinking patterns of sexual minority women appear to be in large part motivated by seeking social connection and a sense of belonging, any intervention developed for sexual minority women must address these social and affective needs [59-61]. An intervention that incorporates these elements within sober curiosity, a wellness movement that challenges the centrality of alcohol in social life and questions the notion that alcohol is required on all social occasions [62], offers a potentially scalable approach to alcohol interventions suitable for sexual minority women. Supported by popular and well-known periods of nondrinking such as “Dry January” or “Sober October,” sober curiosity is geared toward developing longer-term, sustainable lower-risk drinking practices.

#### Potential Usefulness of an Intervention Based on Sober Curiosity

There is evidence of substantive social change regarding alcohol consumption, including the exponential rise in the marketing and availability of no or low alcohol (referred to as “NoLo”) content wines, beers, and spirits [63,64] and a growing number of sober venues and events, as well as proliferation of web-based support forums that are based on sober curiosity tenets. For example, Grace [65], author of *This Naked Mind* and founder of The Alcohol Experiment, reported on February 22, 2023, that in less than 1 year, more than 80,000 people globally had subscribed to the This Naked Mind web-based alcohol reduction program; notably, most subscribers are women. Similarly, Club Soda in the United Kingdom reportedly has helped “tens of thousands” of people reduce their harmful drinking.

The sober curious movement encourages individuals to question the centrality of drinking alcohol in their daily lives, to make a conscious decision to “be curious” about their reasons for drinking, and to consider if drinking alcoholic beverages is necessary in situations where drinking would typically occur. The growing popularity of sober curiosity provides support for a more positive identity for light or nondrinkers than has been previously available. It offers opportunities for both personal and social change, recognizing that individual alcohol consumption behaviors cannot be separated from their contexts. However, no research has examined how sober curiosity or similar interventions to reduce alcohol consumption are perceived by sexual minority women. An established literature on the links between minority stress and poor health [66-68] provides strong evidence that the contexts in which sexual minority women live and experience life differ in important ways (eg, marginalized identities and shame) from heterosexual women’s life experiences, making it critically important that such experiences be considered in any intervention effort to reduce sexual minority women’s alcohol consumption.

Drawing on several decades of our own and others’ research investigating and seeking to understand sexual minority women’s drinking, we believe that a sober curious approach may increase the desire of sexual minority women to reduce alcohol consumption and increase their self-efficacy to resist drinking. Sexual minority women typically participate in alcohol-centric social contexts (social worlds) to connect with other sexual minority people, establish inclusive friendships with like-minded people, and gain social supports that are limited in other contexts [59,60]. These social worlds, although contoured by heavy drinking norms, can be especially helpful during periods of transition or stressful events (eg, coming out, experiencing violence, or relationship dissolution), contexts in which sexual minority women may be more likely to drink heavily [69].

Sober curious approaches could promote wider availability of alternatives that meet important personal and interpersonal needs but do not involve drinking alcohol. This alternative is promising, given research that has found social norms, particularly perceived peer-drinking norms, are strongly predictive of heavy drinking among sexual minority women [32,70,71]. Sober curiosity focuses on shifting cultural and subcultural heavy-drinking norms (not just individual behavior), and interventions that are shaped around the philosophy of sober curiosity may be an important way to address persistent sexual orientation–related disparities in hazardous drinking among women. Sober curiosity is nonjudgmental; it emphasizes alcohol and its addictive properties rather than the person as the “problem.” Research across various domains of sexual minority women’s health suggests that approaches that are nonpathologizing and nonstigmatizing, that focus on community norms and social connection, and that emphasize health, wellness, and mindfulness are likely to be most effective for sexual minority women [72]. Yet, no studies have been conducted that use these approaches in conjunction with sober curious principles to address sexual minority women’s drinking.

Therefore, we are conducting 2 studies that combined to use a variety of methods to explore the feasibility, potential usefulness, and key elements of a tailored intervention based on sober curiosity for sexual minority women. One of the studies includes focus groups and a national survey with sexual minority women in Scotland; the other includes in-depth interviews with sexual minority women in the United States, followed by a review of findings by 2 international panels of experts. The aims of this research are (1) to explore the perspectives of the drinking social worlds of sexual minority women, their awareness of the sober curious movement, perceptions of their own and their peers’ drinking and desire to drink less, and perceived barriers and facilitations to changing their drinking behaviors and (2) to identify key elements of an alcohol reduction intervention tailored for sexual minority women.

#### Conceptual Framework

The conceptual framework informing our work incorporates “social world” perspectives [58] and tenants of the health equity framework [73]. The social worlds of heavy-drinking sexual minority women are characterized by a connection point that can be thought of as a space where drinking alcohol is perceived to be an important aspect of shared activities or of a shared culture [3]. Research addressing heavy drinking social worlds is nascent; yet, this perspective can provide a unique lens for understanding and addressing harmful drinking in at-risk populations. The health equity framework builds on 3 foundational concepts: equity at the core of health outcomes; multiple, interacting spheres of influence; and historical and life-course perspectives. It illustrates how complex interactions between people and their environments affect health behaviors and outcomes. The importance of each of these concepts for LGBTQ+ people is highlighted in a 2011 landmark report on LGBT health [74].

### Why Scotland and the United States?

#### Scotland

Most sexual and gender minority health research, especially research on alcohol use, has been conducted in North America [14,75-78]. We chose to collect data from sexual minority women in Scotland because the sober curiosity movement appears to be more prominent in the United Kingdom than in other European countries and in most other parts of the world [79,80]. Further, hazardous drinking in the UK general population is more prevalent than in other countries in continental Europe [81], and alcohol-related harm is disproportionately higher in Scotland than in the rest of the United Kingdom [82]. In 2018, as part of a raft of evidence-based strategies to tackle this “alcohol crisis,” Scotland became one of the first countries in the world to implement minimum unit pricing with the aim of reducing alcohol-related deaths and decreasing health care use [83,84]. Although relatively little research on alcohol use among sexual and gender minority people has been conducted in Scotland, or in the United Kingdom, limited research has documented higher rates of hazardous alcohol use in sexual and gender minority people in the United Kingdom relative to their heterosexual counterparts [85]. Thus, although no studies have compared rates of sexual and gender minority drinking in Scotland and in the United States, there is reason to believe that sexual minority women in each country are at heightened risk of harmful alcohol use. Our research aims to understand similarities and differences in the cultural contexts of sexual minority women’s drinking in the 2 countries and how such differences impact sexual minority women’s drinking.

#### The United States

Because Scotland’s population is predominately White (only 4% are Asian, African, Caribbean or Black, mixed, or members of other ethnic groups) [86] and because we wanted to include diverse sexual minority women’s perspectives, we chose to also collect data in the United States. We created a sampling frame consisting of diverse sexual minority women in regard to age, sexual and gender identity, race or ethnicity, education, and geographic residence from participants enrolled in an ongoing longitudinal study of risk and protective factors for hazardous drinking among sexual minority women [87].

## Methods

### Study Design

**Figure 1:**
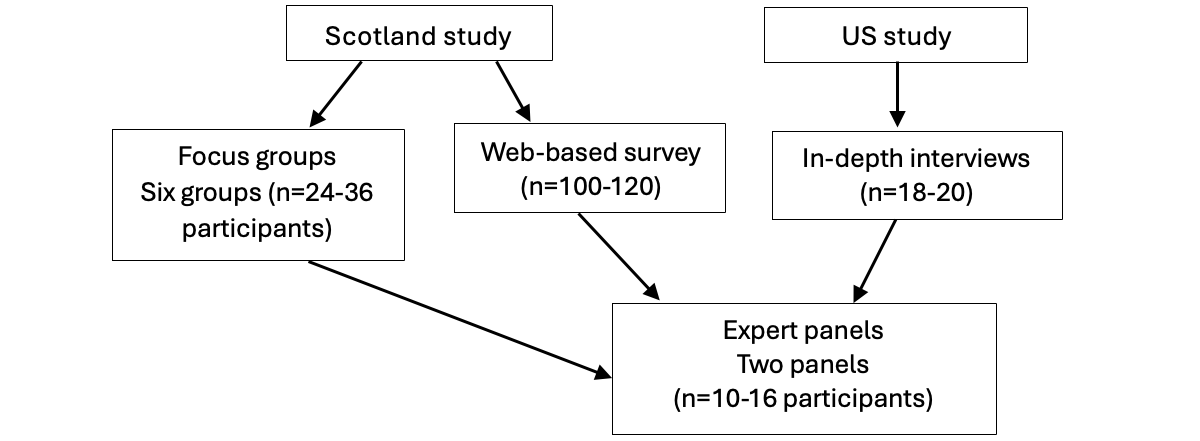
Overview of research components.

We are currently conducting focus groups and a national web-based survey with sexual minority women in Scotland and in-depth individual interviews with sexual minority women in the United States. This mixed methods or triangulated approach, using both qualitative (focus group and individual interviews) and quantitative (web-based survey) methods conducted in 2 countries, was intentionally chosen to maximize understanding of similarities and differences in cultural contexts and influences on sexual minority women’s drinking. Triangulation is commonly used in both qualitative and quantitative research and is especially helpful in understanding complex human behavior. Triangulating methods and data can enhance the validity and credibility of findings, mitigate potential biases, and give more confidence in the findings [88]. Figure 1 summarizes the qualitative and quantitative components of the studies and includes the planned number of participants in each component of the studies.

### Focus Groups

The Scotland study will include 5 to 6 focus groups, each with a purposive volunteer sample of 4 to 6 adult sexual minority women. Focus group interviews are conducted in person or via Zoom (Zoom Video Communications) and are led by team members (primarily LB and BM) who are experienced qualitative researchers with expertise in alcohol use among sexual minority women; some team members identify as part of the sexual minority women’s community. Focus group discussions are organized around specific topics and include broad areas of inquiry [89] developed by the research team. The focus group interview guide was adapted following the first and second focus group sessions to improve clarity and more effectively address the study aims. Areas of inquiry include (1) perceptions of alcohol use in sexual minority women’s “communities” in Scotland; (2) openness to reducing alcohol consumption (are they sober curious?); (3) factors and barriers that impact sexual minority women’s decisions and preparedness to reduce consumption (eg, drinking norms and drinking behaviors of peers); (4) attitudes toward, perceived availability of, and experiences with NoLo alcohol beverage alternatives; (5) conditions that would support reducing their own and other sexual minority women’s alcohol consumption; (6) messaging and framing about reducing alcohol consumption that sexual minority women would find appealing (or off-putting); and (7) the potential benefits and barriers to a successful sober curious alcohol reduction intervention with sexual minority women. The focus group interview guide is available upon request.

### Web-Based Survey

We will complement data collected in focus groups with data collected from a larger convenience sample of Scottish sexual minority women (N=100-120) recruited to participate in a web-based Qualtrics survey. Our team developed the survey using existing literature and a previous study on sober curiosity conducted by Lunnay et al [90] with midlife women in Australia. We pilot-tested the survey with 10 sexual minority women in Scotland and the United States prior to its official launch. The survey includes approximately 80 questions (3-5 questions per page) and takes an average of 15-20 minutes to complete. Like focus groups, the survey includes questions about demographic characteristics, patterns of alcohol use (eg, frequency and quantity), drinking contexts, drinking companions, drinking motivations and perceived drinking norms of peers, as well as help-seeking, familiarity with the sober curious movement, or aspects of it (eg, Dry January, Sober October, and NoLo alcohol beverages), and perceived barriers and facilitators to reducing alcohol consumption. We also include a few open-ended questions to collect qualitative data that will be used to triangulate data from the focus groups (eg, participants’ descriptions of barriers and facilitators to reducing drinking).

### Scotland Study Recruitment

We are recruiting adult (age 18 years or older) sexual minority women who reside in Scotland and who report moderate (between 7 and 14 units per week) or heavy (>14 units per week) alcohol consumption. Although there is no agreed international definition of low- or moderate-risk drinking [91,92], researchers in the United Kingdom [93] have used 7-14 units per week to indicate “moderate” drinking. Efforts to support effective behavior change must consider local sociocultural contexts and build on existing community assets. In each country, we are working to recruit diverse samples of sexual minority women in terms of age, education, geographic location (eg, rural and urban), and identity (eg, lesbian, bisexual, queer, and nonbinary gender). In addition, because sexual minority women’s “communities” are not homogenous, and distinct groups within it are more or less marginalized and have different levels of capacity and resilience than others, we are using recruitment strategies that attract sexual minority women across different sociodemographic statuses. We are posting advertisements in local LGBTQ+ publications, advertising the study via social media and relevant mailing lists, and conducting outreach through partnerships with LGBTQ+ organizations in Scotland (eg, *LGBTQ+ Health & Wellbeing*, *LGBTQ+ Youth-Scotland*, and *The Equality Network*) and LGBTQ+ sports groups. Interested individuals are directed to a web-based screener where they answer several questions about their age, sexual identity, residence, and current drinking to determine eligibility. Prospective participants are informed that, in appreciation of their time and contributions, they will receive an Amazon gift card worth approximately US $38 for participation in a focus group or US $12.40 for completion of the web-based survey.

### US Study

#### In-Depth Interviews

In a separate but complementary study, we are conducting in-depth interviews with a purposive, diverse sample of approximately 18-20 sexual minority women in the United States. Interviews lasting approximately 60 minutes are conducted via Zoom or phone by 2 researchers (LB and Ellen Riggle) with extensive qualitative training and research experience in sexual minority women’s health. Questions explore the same areas of inquiry as the focus groups in Scotland. For example, sexual minority women are asked about their perceptions regarding alcohol use within sexual minority women’s social worlds and about their personal experiences with drinking. During interviews, we ask if sexual minority women are aware of the sober curiosity movement, are interested in considering ways to reduce their alcohol consumption (are they sober curious?), factors and barriers that impact their decisions and preparedness to reduce consumption, alternatives to alcoholic beverages and alcohol-based events perceived to be available, attitudes toward and experiences with NoLo beverages, and perceptions of conditions that would support consideration of reducing their own and other sexual minority women’s alcohol consumption. The US interview guide is available upon request.

#### Recruitment for US In-Depth Interviews

We are recruiting adult sexual minority women who participated in a longitudinal study of drinking among sexual minority women led by first author Hughes [14, 94]. This approach permits more efficient and accurate identification of sexual minority women who either drink at moderate or heavy levels (ie, at least 14 drinks per week on average) or reported that they were thinking of cutting down on their alcohol use. The sample will include sexual minority women with diverse demographic characteristics, such as age, race or ethnicity, sexual identity (eg, lesbian, bisexual, and queer), residence (urban or rural), educational level, and relationship status. Interviews last 60-90 minutes, and participants are given a US $50 Amazon gift card in appreciation of their time.

### Ethical Considerations

The Scotland study received ethics approval from Glasgow Caledonian University (HSL NCH 22 040), and both the Scotland and the US studies received ethics approval from Columbia University (AAAU7672), the lead institution. The studies were reviewed and deemed to be exempt given that they were judged to pose little or no risk to the research participants. For the Scotland focus groups, informed consent is obtained by the researchers prior to commencing research procedures. An information sheet is provided to individuals who participate in the web-based survey with passive consent implied by virtue of survey completion. For the US study, verbal informed consent is obtained by the project manager prior to scheduling each participant’s interview. Following a verbal review of the consent form, questions are asked of each participant to ascertain that they understand what is involved in their participation. Documentation of consent is maintained on a password-protected end-point device. Participants receive a copy of the consent form via email for their records. Scotland focus groups are conducted either in person or on Zoom (to accommodate the participation of sexual minority women who live in rural or other outlying areas). Participants are invited to provide a pseudonym to be used in focus group discussions, and any names or information that could potentially identify a participant are redacted from the transcripts. Each focus group participant is assigned a unique ID number linked to their demographic and contact details, which are saved separately on a password-protected system in a password-protected file. Audio files are destroyed after the transcripts are reviewed and verified. Deidentified transcripts will be available to members of the research team for analyses. Scotland survey participants are also each assigned a unique ID number and are asked to provide an email address in order to receive the link for the study incentive (Amazon gift card). The key that links ID numbers to email addresses is kept on a password-protected system in a password-protected file separate from the dataset with the survey responses. The ID numbers are used to keep track of who has been sent a gift card. Individual interviews in the United States are conducted by phone or Zoom. Audio files are coded with the participant’s identification number but without any information that could be used to identify them. The list that links codes to names and addresses is kept on a password-protected end-point device in a password-protected file separate from the audio files. Once interviews are completed, the audio files of the interviews are saved on a secure electronic system (ie, Box). Once the interview transcripts are reviewed and verified, the audio files will be destroyed.

### Data Analysis

Given the range of data sources used, it is essential to triangulate data to address the completeness, convergence, and dissonance of key themes. We will analyze data from each of the 3 sources separately.

Data from the focus groups (Scotland) will be analyzed using Krueger’s framework analysis [95] for narrative focus group data that considers words, context, internal consistency, frequency of comments, extensiveness of comments, intensity of comments, specificity of responses, and big ideas. This process involves six interconnected steps: (1) data collection; (2) immersion in data; (3) memoing to identify ideas and concepts; (4) indexing descriptive statements; (5) formation of categories, drawing on within- and between-group comparisons; and (6) interpretation. In-depth interview data from the US study will be analyzed using inductive thematic analysis drawing on six phases described by Braun and Clarke [96]: (1) immersion through reading and rereading data and noting initial ideas, (2) generating initial codes, (3) searching for themes, (4) reviewing themes, (5) defining and naming themes, and (6) producing the report, including selection and use of extract examples. Thematic analysis is a method for identifying and organizing patterns of meaning, or themes, across a dataset to make sense of shared meaning and collective experiences [97]. Analyses of the Scotland and US qualitative data will be guided by a social worlds’ perspective, which aligns with constructivist and symbolic interaction theory and “supports identification and investigation of groups below the level of society but above the level of friendship groups, where relatively coherent heavy drinking cultures may operate” [3].

Although data will be analyzed separately, we will be particularly interested in comparing themes from the focus groups and the in-depth interviews to identify similarities and differences in the findings and to identify patterns and relationships across data to validate or corroborate findings. We expect that these comparisons will provide a more comprehensive understanding of factors that serve as facilitators or barriers to sexual minority women who wish to reduce their alcohol intake.

Several strategies will be used to enhance the trustworthiness of the qualitative findings [98]. These include prolonged engagement with the narrative data, researcher triangulation, with multiple researchers each bringing their perspective to analyses, and regular peer debriefing and reflexive meetings via Zoom with research team members (authors of this paper). The team members are part of a research collaboration among Columbia University School of Nursing, Center for Sexual and Gender Minority Health Research and international partners at Glasgow Caledonian University, Research Centre for Health, and Torrens University Australia, Research Centre for Public Health, Equity and Human Flourishing. The research team includes individuals from multiple disciplines (eg, public health, sociology, psychology, nursing, and social work). More than half of the team members identify as being part of either sexual minority or gender minority communities. As a check to ensure findings accurately reflect the experiences and perspectives of the participants, member checking will involve validating the findings with stakeholders involved in the research (eg, members of the research team) and with at least 3 focus group participants and 3 participants from the in-depth interview portion of the study.

Survey data will be analyzed using SPSS (version 29; IBM Corp). Analyses will initially screen for and correct outliers, data entry errors, or other inconsistencies. We will decide whether to use cases with incomplete data (eg, surveys that were only partially completed) based on the number and relevance of key questions that have valid responses. All statistical tests, for example, comparing rural versus urban sexual minority women, will be 2-sided (α=.05). Given the exploratory or descriptive nature of the study, we believe that data from 100 to 120 survey participants will provide sufficient information to inform our research aims. We will explore how findings from closed- and open-ended survey questions map onto key themes from the qualitative studies.

Comparisons of the perceptions and experiences of sexual minority women in Scotland and in the United States will identify thematic commonalities and differences and to formulate hypotheses about how drinking alcohol among sexual minority women relates to social contexts, group identity, and cultural norms. Although we expect to find similarities in the cultural contexts of sexual minority women across these 2 high-income countries, understanding differences and how they shape drinking norms and behaviors will elucidate key elements of a future tailored intervention aimed at helping sexual minority women reduce the risk of harmful drinking.

## Results

The study was funded in July 2023, and data collection began in September 2023 after the study received ethics approval from the 2 lead institutions (August 2023). The Scotland study received ethics approval from Glasgow Caledonian University (HSL NCH 22 040) and from Columbia University (AAAU7672). As of December 2024, we completed 4 of the 6 planned focus groups (Scotland) and 18 of the 20 planned in-depth interviews in the United States. Data collection for the web-based survey in Scotland was paused in October 2024 for several months after discovering that about two-thirds of the approximately 150 responses were invalid (exhibited evidence of bot automation, eg, duplicate IP addresses). To address this, we removed the Amazon gift card incentive from recruitment advertisements and submitted the revised protocol to the 2 human participant review committees for ethical approval. As of December 2024, we have 64 valid survey responses. We expect to complete data collection in both Scotland and the United States by June 2025.

Results of qualitative analyses (data from focus groups and individual interviews) will be organized and summarized as themes, and results of survey data analyses summarized in tables. Findings from the studies will be presented to 2 international panels of experts; each panel will include 4-6 experts. Panel participants will consist of researchers with expertise in sexual minority women’s use of alcohol, the development of alcohol interventions for women, or sober curiosity approaches. This will serve 2 purposes: peer review (having other researchers or experts review the findings to ensure their validity and to identify potential biases) and identification of key factors to be included in a planned intervention that will incorporate tenants of sober curiosity. We will use Acceptability, Practicability, Effectiveness, Affordability, Side-Effects, and Equity [99] criteria to consider important socioecological and cultural factors that provide an overarching understanding of key elements of an intervention tailored to assist sexual minority women to reduce harmful drinking.

## Discussion

### Principal Findings

To our knowledge, this project is the first attempt to investigate sexual minority women’s openness and readiness to reduce alcohol consumption within a sober curiosity framework and with the view to designing an intervention tailored for sexual minority women. Understanding how sexual minority women might justify or rationalize their alcohol consumption in different contexts and social worlds, their interest in changing their drinking behaviors to reduce their alcohol consumption, and their perceived barriers and facilitators to doing so will provide new insights. We anticipate that findings will provide essential preliminary data to develop (and subsequently evaluate) an intervention that incorporates tenets of the sober curious movement and is aimed at reducing harmful drinking among sexual minority women, with scalability to other high-income countries with similar drinking-related social norms. Such an intervention will respond to sexual minority women’s reported need for social connection and will support alcohol reduction strategies while retaining social support.

### Strengths and Limitations

First, the use of mixed methods to collect data, including both qualitative and quantitative methods, and the inclusion of 2 countries are strengths of this research. For example, while the perspectives of sexual minority women about alcohol use and sober curiosity in the web-based survey may be influenced by misunderstandings of the questions, focus group and individual interviews allow for such misunderstandings to be explored and corrected. However, relationships among drinking behaviors and patterns, sexuality, and gender are complex [5,30,60,100], and information to be gleaned from the current research may not capture these complexities. For example, qualitative research from Scotland and Australia highlights the ways in which drinking alcohol facilitates engagement with LGBTQ+ communities, aids in expressing sexuality and identity, and challenges heteronormative perspectives of women’s drinking [30,100]. Our focus on sober curiosity and ways that sexual minority women might reduce their alcohol consumption does not consider the performative role that alcohol use, including heavier use, plays in “doing” gender and sexuality and in connecting with the LGBTQ+ community in pleasurable and meaningful ways. Future work should consider the complex meanings and social functions of alcohol in the lives of sexual minority women.

Second, because findings are from nonrepresentative samples of sexual minority women in Scotland and in the United States, they have limited transferability. Both countries are characterized by high income and relatively low levels of stigma (eg, compared to non-Western and low-income countries). There may be important cultural differences that shape alcohol use among sexual minority women who live in countries and cultural contexts where either same-sex sexuality or alcohol use among women is more stigmatized. Further, given limited time and resources, we may not be able to reach as diverse a sample as intended. For example, factors such as poverty and homelessness or disability influence sexual minority women’s drinking. More research using mixed methods and larger samples across different cultural contexts is needed to further refine the understanding of factors that influence sexual minority women’s drinking and perspectives about sober curiosity.

Third, although trauma is not a focus of the studies, it is well documented that experiences of lifetime trauma, particularly adverse childhood experiences, are more prevalent among sexual minority women [24] and are strongly linked with alcohol use among sexual minority women [14,101]. Thus, our failure to address trauma may limit important insights about key elements of an alcohol reduction intervention for sexual minority women.

### Conclusions

The sober curious movement is gaining positive attention and momentum in mainstream society in many parts of the world. Now is an important time to leverage this momentum to address heavy drinking social norms and behaviors within sexual minority women’s social worlds. An intervention developed and offered to sexual minority women within this growing movement may provide an acceptable and effective way to support sexual minority women to reduce alcohol-related harms. However, such interventions will likely need to recognize the positive aspects of drinking (eg, facilitating social connections) and the pervasive impact of trauma on sexual minority women’s drinking as well as the linkages among connection, coping, and identity, if they are to be optimally effective.

## Abbreviations

AA: Alcoholics Anonymous
CBT: cognitive behavioral therapy
LGB: lesbian, gay, and bisexual
LGBTQ+: lesbian, gay, bisexual, transgender, queer or questioning
WHO: World Health Organization

## Data Availability

The datasets generated and analyzed as part of this project will be available from the corresponding author on reasonable request after the research team has completed analyses related to the study aims.

## References

[1] Reid MC, Fiellin DA, O'Connor PG (1999). Hazardous and harmful alcohol consumption in primary care. Arch Intern Med.

[2] (2024). Alcohol: fact sheet. World Health Organization.

[3] MacLean S, Dwyer R, Pennay A, Savic M, Wilkinson C, Roberts S, Turner K, Saleeba E, Room R (2020). The ‘social worlds’ concept: a useful tool for public health-oriented studies of drinking cultures. Addict Res Theory.

[4] (2018). Global status report on alcohol and health 2018. World Health Organization.

[5] Grant BF, Chou SP, Saha TD, Pickering RP, Kerridge BT, Ruan WJ, Huang B, Jung J, Zhang H, Fan A, Hasin DS (2017). Prevalence of 12-month alcohol use, high-risk drinking, and DSM-IV alcohol use disorder in the United States, 2001-2002 to 2012-2013: results from the National Epidemiologic Survey on Alcohol and Related Conditions. JAMA Psychiatry.

[6] Davey C (2021). Online sobriety communities for women's problematic alcohol use: a mini review of existing qualitative and quantitative research. Front Glob Womens Health.

[7] Miller M, Mojica-Perez Y, Livingston M, Kuntsche E, Wright CJC, Kuntsche S (2022). The who and what of women's drinking: examining risky drinking and associated socio-demographic factors among women aged 40-65 years in Australia. Drug Alcohol Rev.

[8] Meng Y, Holmes J, Hill-McManus D, Brennan A, Meier PS (2014). Trend analysis and modelling of gender-specific age, period and birth cohort effects on alcohol abstention and consumption level for drinkers in Great Britain using the General Lifestyle Survey 1984-2009. Addiction.

[9] Greene N, Jackson JW, Dean LT (2020). Examining disparities in excessive alcohol use among Black and Hispanic lesbian and bisexual women in the United States: an intersectional analysis. J Stud Alcohol Drugs.

[10] Lunnay B, Foley K, Meyer SB, Warin M, Wilson C, Olver I, Miller ER, Thomas J, Ward PR (2021). Alcohol consumption and perceptions of health risks during COVID-19: a qualitative study of middle-aged women in South Australia. Front Public Health.

[11] Lunnay B, Toson B, Wilson C, Miller ER, Meyer SB, Olver IN, Foley K, Thomas JA, Ward PR (2021). Social class and changes in Australian women's affect and alcohol consumption during COVID-19. Front Public Health.

[12] Miller ER, Olver IN, Wilson CJ, Lunnay B, Meyer SB, Foley K, Thomas JA, Toson B, Ward PR (2021). COVID-19, alcohol consumption and stockpiling practises in midlife women: repeat surveys during lockdown in Australia and the United Kingdom. Front Public Health.

[13] Bloomfield K (2020). Understanding the alcohol-harm paradox: what next?. Lancet Public Health.

[14] Hughes TL, Veldhuis CB, Drabble LA, Wilsnack SC (2020). Research on alcohol and other drug (AOD) use among sexual minority women: a global scoping review. PLoS One.

[15] Mulia N, Bensley KM (2020). Alcohol-related disparities among women: evidence and potential explanations. Alcohol Res.

[16] Hughes TL, Wilsnack SC, Kantor LW (2016). The influence of gender and sexual orientation on alcohol use and alcohol-related problems: toward a global perspective. Alcohol Res.

[17] Fish JN, Hughes TL, Russell ST (2018). Sexual identity differences in high-intensity binge drinking: findings from a US national sample. Addiction.

[18] Dinos S, Tay N, Shipsey F, Neave A (2021). Health and Health-Related Behaviours of Lesbian, Gay and Bisexual Adults.

[19] Dyar C, Dworkin ER, Pirog S, Kaysen D (2021). Social interaction anxiety and perceived coping efficacy: mechanisms of the association between minority stress and drinking consequences among sexual minority women. Addict Behav.

[20] Cerezo A, Williams C, Cummings M, Ching D, Holmes M (2019). Minority stress and drinking: connecting race, gender identity and sexual orientation. Couns Psychol.

[21] Lewis RJ, Romano KA, Ehlke SJ, Lau-Barraco C, Sandoval CM, Glenn DJ, Heron KE (2021). Minority stress and alcohol use in sexual minority women's daily lives. Exp Clin Psychopharmacol.

[22] Porsch LM, Xu M, Veldhuis CB, Bochicchio LA, Zollweg SS, Hughes TL (2023). Intimate partner violence among sexual minority women: a scoping review. Trauma Violence Abuse.

[23] Bochicchio L, Xu M, Veldhuis CB, McTavish C, Hughes TL (2023). Mental health and substance use among sexual minority women who report childhood sexual abuse: a systematic literature review. Psychol Trauma.

[24] Bochicchio L, Porsch L, Zollweg S, Matthews AK, Hughes TL (2024). Health outcomes of sexual minority women who have experienced adverse childhood experiences: a scoping review. Trauma Violence Abuse.

[25] Wilsnack SC, Kristjanson AF, Hughes TL, Benson PW (2012). Characteristics of childhood sexual abuse in lesbians and heterosexual women. Child Abuse Negl.

[26] Hughes TL, Johnson TP, Steffen AD, Wilsnack SC, Everett B (2014). Lifetime victimization, hazardous drinking, and depression among heterosexual and sexual minority women. LGBT Health.

[27] Alvy LM, Hughes TL, Kristjanson AF, Wilsnack SC (2013). Sexual identity group differences in child abuse and neglect. J Interpers Violence.

[28] Scheer JR, Helminen EC, Cascalheira CJ, Jaipuriyar V, Shaw TJ, Zabelski S, Behari K, Pirog S, Batchelder AW, Possemato K, Hughes TL, Sullivan TP (2023). Probable PTSD, PTSD symptom severity, and comorbid PTSD and hazardous drinking among sexual minority women compared to heterosexual women: a meta-analysis. Clin Psychol Rev.

[29] Whiteley D, Rickards-Hill D, Dimova E, Emslie C (2023). Performing solidarity? A scoping review of alcohol marketing to sexual and gender minorities. Drugs Educ Prev Policy.

[30] Emslie C, Lennox J, Ireland L (2017). The role of alcohol in identity construction among LGBT people: a qualitative study. Sociol Health Illn.

[31] MacLean S, Savic M, Pennay A, Dwyer R, Stanesby O, Wilkinson C (2018). Middle-aged same-sex attracted women and the social practice of drinking. Critical Public Health.

[32] Boyle SC, LaBrie JW, Omoto AM (2020). Normative substance use antecedents among sexual minorities: a scoping review and synthesis. Psychol Sex Orientat Gend Divers.

[33] Dworkin ER, Cadigan J, Hughes T, Lee C, Kaysen D (2018). Sexual identity of drinking companions, drinking motives, and drinking behaviors among young sexual minority women: an analysis of daily data. Psychol Addict Behav.

[34] Gilbert PA, Pro G, Zemore SE, Mulia N, Brown G (2019). Gender differences in use of alcohol treatment services and reasons for nonuse in a national sample. Alcohol Clin Exp Res.

[35] Batchelder AW, Stanton AM, Kirakosian N, King D, Grasso C, Potter J, Mayer KH, O'Cleirigh C (2021). Mental health and substance use diagnoses and treatment disparities by sexual orientation and gender in a community health center sample. LGBT Health.

[36] Krasnova A, Diaz JE, Philbin MM, Mauro PM (2021). Disparities in substance use disorder treatment use and perceived need by sexual identity and gender among adults in the United States. Drug Alcohol Depend.

[37] Dimova ED, O'Brien R, Elliott L, Frankis J, Emslie C (2022). Exploring the experiences of alcohol service use among LGBTQ+ people in Scotland: a qualitative study. Int J Drug Policy.

[38] Cascalheira CJ, Helminen EC, Shaw TJ, Scheer JR (2022). Structural determinants of tailored behavioral health services for sexual and gender minorities in the United States, 2010 to 2020: a panel analysis. BMC Public Health.

[39] Scheer JR, Batchelder AW, Bochicchio LA, Kidd JD, Hughes TL (2022). Alcohol use, behavioral and mental health help-seeking, and treatment satisfaction among sexual minority women. Alcohol Clin Exp Res.

[40] Kidd JD, Paschen-Wolff MM, Mericle AA, Caceres BA, Drabble LA, Hughes TL (2022). A scoping review of alcohol, tobacco, and other drug use treatment interventions for sexual and gender minority populations. J Subst Abuse Treat.

[41] Dimova ED, Elliott L, Frankis J, Drabble L, Wiencierz S, Emslie C (2022). Alcohol interventions for LGBTQ+ adults: a systematic review. Drug Alcohol Rev.

[42] Sanders JM (2020). Seeking acceptance: LGBTQ and membership in Alcoholics Anonymous (AA). Alcohol Treat Q.

[43] McGeough BL, Karriker-Jaffe KJ, Zemore SE (2021). Rates and predictors of Alcoholics Anonymous attendance across sexual orientations. J Subst Abuse Treat.

[44] Hall JM (1994). The experiences of lesbians in Alcoholics Anonymous. West J Nurs Res.

[45] Senreich E (2009). Demographic, background, and treatment factors that affect gay and bisexual clients in substance abuse programs. J LGBT Iss Couns.

[46] Bernier LB, Foley JD, Salomaa AC, Scheer JR, Kelly J, Hoeppner B, Batchelder AW (2024). Examining sexual minority engagement in recovery community centers. J Subst Use Addict Treat.

[47] Paschen-Wolff MM, DeSousa A, Paine EA, Hughes TL, Campbell ANC (2024). Experiences of and recommendations for LGBTQ+-affirming substance use services: an exploratory qualitative descriptive study with LGBTQ+ people who use opioids and other drugs. Subst Abuse Treat Prev Policy.

[48] McGeough BL, Zemore SE, Dastur Z, Neilands TB, Lisha NE, Lunn MR, Obedin-Maliver J, Lubensky ME, Flentje A (2024). Levels and outcomes of 12-step participation among sexual and gender minority subgroups. J Subst Use Addict Treat.

[49] Drabble LA, Mericle AA, Munroe C, Cerezo A, Karriker-Jaffe KJ, Hughes TL, Trocki KF (2022). Examining the differential protective effects of women's spirituality and religiosity on alcohol and marijuana use by sexual identity. Addict Behav Rep.

[50] Drabble L, Veldhuis CB, Riley BB, Rostosky S, Hughes TL (2018). Relationship of religiosity and spirituality to hazardous drinking, drug use, and depression among sexual minority women. J Homosex.

[51] Dillworth TM, Kaysen D, Montoya HD, Larimer ME (2009). Identification with mainstream culture and preference for alternative alcohol treatment approaches in a community sample. Behav Ther.

[52] Zajac K, Rash CJ, Ginley MK, Heck NC (2020). Sexual orientation and substance use treatment outcomes across five clinical trials of contingency management. Psychol Addict Behav.

[53] Gilmore AK, Leone RM, Oesterle DW, Davis KC, Orchowski LM, Ramakrishnan V, Kaysen D (2022). Web-based alcohol and sexual assault prevention program with tailored content based on gender and sexual orientation: preliminary outcomes and usability study of positive change (+Change). JMIR Form Res.

[54] Fals-Stewart W, O'Farrell TJ, Lam WKK (2009). Behavioral couple therapy for gay and lesbian couples with alcohol use disorders. J Subst Abuse Treat.

[55] Pachankis JE, McConocha EM, Clark KA, Wang K, Behari K, Fetzner BK, Brisbin CD, Scheer JR, Lehavot K (2020). A transdiagnostic minority stress intervention for gender diverse sexual minority women's depression, anxiety, and unhealthy alcohol use: a randomized controlled trial. J Consult Clin Psychol.

[56] Boyle SC, LaBrie JW, Trager BM, Costine LD (2022). A gamified personalized normative feedback app to reduce drinking among sexual minority women: randomized controlled trial and feasibility study. J Med Internet Res.

[57] Bush R, Staiger PK, McNeill IM, Brown R, Orellana L, Lubman D, McNair R (2024). Evaluation of an SMS based alcohol intervention for same sex attracted women: a randomized controlled trial to examine feasibility, acceptability, and efficacy. Subst Use Misuse.

[58] Room R, MacLean S, Pennay A, Dwyer R, Turner K, Saleeba E (2021). Changing risky drinking practices in different types of social worlds: concepts and experiences. Drugs Educ Prev Policy.

[59] Condit M, Kitaji K, Drabble L, Trocki K (2011). Sexual minority women and alcohol: intersections between drinking, relational contexts, stress and coping. J Gay Lesbian Soc Serv.

[60] McNair R, Pennay A, Hughes T, Brown R, Leonard W, Lubman DI (2016). A model for lesbian, bisexual and queer-related influences on alcohol consumption and implications for policy and practice. Cult Health Sex.

[61] Emslie C, Lennox J, Ireland L (2015). The social context of LGBT people's drinking in Scotland. Scottish Health Action on Alcohol Problems.

[62] Warrington R, Winther R (2018). Sober Curious.

[63] Dingwall K (2022). The no-alcohol drinks market surpassed $11 billion in 2022. Forbes.

[64] (2020). New report reveals key features of no- and low-alcohol drinks market. National Institute for Health and Care Research.

[65] Grace A (2023). The Alcohol Experiment. This Naked Mind.

[66] Meyer IH (2003). Prejudice, social stress, and mental health in lesbian, gay, and bisexual populations: conceptual issues and research evidence. Psychol Bull.

[67] Brooks VR (1982). Minority Stress and Lesbian Women.

[68] Hatzenbuehler ML (2009). How does sexual minority stigma "get under the skin"? A psychological mediation framework. Psychol Bull.

[69] Trocki K, Drabble L (2008). Bar patronage and motivational predictors of drinking in the San Francisco Bay area: gender and sexual identity differences. J Psychoactive Drugs.

[70] Ehlke SJ, Stamates AL, Kelley ML, Braitman AL (2019). Bisexual women's reports of descriptive drinking norms for heterosexual, bisexual, and lesbian women. Psychol Sex Orientat Gend Divers.

[71] Cochran SD, Grella CE, Mays VM (2012). Do substance use norms and perceived drug availability mediate sexual orientation differences in patterns of substance use? Results from the California Quality of Life Survey II. J Stud Alcohol Drugs.

[72] Garbers S, McDonnell C, Fogel SC, Eliason M, Ingraham N, McElroy JA, Radix A, Haynes SG (2015). Aging, weight, and health among adult lesbian and bisexual women: a metasynthesis of the multisite "Healthy Weight Initiative" focus groups. LGBT Health.

[73] Peterson A, Charles V, Yeung D, Coyle K (2021). The health equity framework: a science- and justice-based model for public health researchers and practitioners. Health Promot Pract.

[74] Institute of Medicine (US) Committee on Lesbian, Bisexual, and Transgender Health Issues and Research Gaps and Opportunities (2011). The Health of Lesbian, Gay, Bisexual, and Transgender People: Building a Foundation for Better Understanding.

[75] Gilbert PA, Pass LE, Keuroghlian AS, Greenfield TK, Reisner SL (2018). Alcohol research with transgender populations: a systematic review and recommendations to strengthen future studies. Drug Alcohol Depend.

[76] King M, Semlyen J, Tai SS, Killaspy H, Osborn D, Popelyuk D, Nazareth I (2008). A systematic review of mental disorder, suicide, and deliberate self harm in lesbian, gay and bisexual people. BMC Psychiatry.

[77] Pellicane MJ, Quinn ME, Ciesla JA (2023). Transgender and gender-diverse minority stress and substance use frequency and problems: systematic review and meta-analysis. Transgender Health.

[78] Chapa Montemayor AS, Connolly DJ (2023). Alcohol reduction interventions for transgender and non-binary people: a PRISMA-ScR-adherent scoping review. Addict Behav.

[79] Atkinson AM, Matthews BR, Nicholls E, Sumnall H (2023). 'Some days I am a lunatic that thinks I can moderate': amalgamating recovery and neo-liberal discourses within accounts of non-drinking among women active in the 'positive sobriety' community on instagram in the UK. Int J Drug Policy.

[80] Atkinson AM, Meadows BR, Sumnall HR (2022). ‘You’re in the alcohol Matrix, then you unplug from it, and you’re like ‘Wow’’’: exploring sober women’s management, negotiation and countering of alcohol marketing in the UK. Drugs Educ Prev Policy.

[81] Winstock A, Maier LJ, Zhuparris A, Davies E, Puljevic C, Kuypers K, Ferris JA, Barratt MJ (2021). Global Drug Survey: GDS 2021.

[82] McCartney G, Bouttell J, Craig N, Craig P, Graham L, Lakha F, Lewsey J, McAdams R, MacPherson M, Minton J, Parkinson J, Robinson M, Shipton D, Taulbut M, Walsh D, Beeston C (2016). Explaining trends in alcohol-related harms in Scotland, 1991-2011 (I): the role of incomes, effects of socio-economic and political adversity and demographic change. Public Health.

[83] MacGilchrist A (2023). Scotland's alcohol crisis: a clinician's perspective on a public health emergency. J R Coll Physicians Edinb.

[84] Wyper GMA, Mackay DF, Fraser C, Lewsey J, Robinson M, Beeston C, Giles L (2023). Evaluating the impact of alcohol minimum unit pricing on deaths and hospitalisations in Scotland: a controlled interrupted time series study. Lancet.

[85] Meads C, Zeeman L, Sherriff N, Aranda K (2023). Prevalence of alcohol use amongst sexual and gender minority (LGBTQ+) communities in the UK: a systematic scoping review. Alcohol Alcohol.

[86] (2023). Ethnicity. Scotland's Census.

[87] Hughes TL, Wilsnack SC, Martin K, Matthews AP, Johnson TP (2021). Alcohol use among sexual minority women: methods used and lessons learned in the 20-Year Chicago Health and Life Experiences of Women Study. Int J Alcohol Drug Res.

[88] Noble H, Heale R (2019). Triangulation in research, with examples. Evid Based Nurs.

[89] Morgan DL, Krueger RA (1998). Developing Questions for Focus Groups.

[90] Lunnay B, Nicholls E, Pennay A, MacLean S, Wilson C, Meyer SB, Foley K, Warin M, Olver I, Ward PR (2022). Sober curiosity: a qualitative study exploring women's preparedness to reduce alcohol by social class. Int J Environ Res Public Health.

[91] Dufour MC (1999). What is moderate drinking? Defining "drinks" and drinking levels. Alcohol Res Health.

[92] Furtwaengler NAFF, de Visser RO (2013). Lack of international consensus in low-risk drinking guidelines. Drug Alcohol Rev.

[93] Topiwala A, Allan CL, Valkanova V, Zsoldos E, Filippini N, Sexton C, Mahmood A, Fooks P, Singh-Manoux A, Mackay CE, Kivimäki M, Ebmeier KP (2017). Moderate alcohol consumption as risk factor for adverse brain outcomes and cognitive decline: longitudinal cohort study. BMJ.

[94] Veldhuis CB, Porsch LM, Bochicchio LA, Campbell J, Johnson TP, LeBlanc AJ, Leonard KE, Wall M, Wilsnack SC, Xu M, Hughes TL (2021). The Chicago Health and Life Experiences of Women Couples Study: protocol for a study of stress, hazardous drinking, and intimate partner aggression among sexual minority women and their partners. JMIR Res Protoc.

[95] Krueger RA (1998). Analyzing and Reporting Focus Group Results.

[96] Braun V, Clarke V (2006). Using thematic analysis in psychology. Qual Res Psychol.

[97] Braun V, Clarke V (2020). One size fits all? What counts as quality practice in (reflexive) thematic analysis?. Qual Res Psychol.

[98] Nowell LS, Norris JM, White DE, Moules NJ (2017). Thematic analysis: striving to meet the trustworthiness criteria. Int J Qual Methods.

[99] Charlton J, Gréaux M, Kulkarni A, Dornstauder M, Law J (2024). UK paediatric speech and language therapists' perceptions on the use of telehealth in current and future clinical practice: an application of the APEASE criteria. Int J Lang Commun Disord.

[100] Grant R, Power J, Mooney-Somers J, Pennay A, McNair R, Bourne A (2024). 'All the dykes I know drink beer': sexuality and gender performance through alcohol consumption among lesbian, bisexual, and queer women in Australia. Soc Sci Med.

[101] Hughes T, Szalacha LA, McNair R (2010). Substance abuse and mental health disparities: comparisons across sexual identity groups in a national sample of young Australian women. Soc Sci Med.

